# Supplementary material for: Gastric bypass prevents diabetes in genetically modified mice and chemically induced diabetic mice
Source: PLoS One. 2021 Oct 21;16(10):e0258942. doi: 10.1371/journal.pone.0258942 (PMC8530305; doi:10.1371/journal.pone.0258942)
Supplement: S1 File — (DOCX) [file pone.0258942.s001.docx]

**Mice, diet and surgery**

**Mice.** C57BL/KsJ-db/db nude (BKS-db/db, Cat# 000662, the Jackson Laboratory), New Zealand Obese (NZO, Cat# 004456, the Jackson Laboratory) and C57BL/6-db/db nude (B6-db/db, Cat# 000632, the Jackson Laboratory). Wild-type diabetic mice were induced by intravenous injection of streptozotocin (170mg/kg, Sigma-Aldrich, Cat#: SO130, ≥75% a-anomer basis, ≥98% (HPLC). All mice were housed at 23°C on a 07:00-19:00 light cycle at the Animal Resources Center at the University of Chicago.

**Rodent Chow Diet Information.** Certified ingredient composition can be found at Mazuri (Formula Code - 5663).

Protein 23%; Fat ≥ 6.5%; Crude fiber not more than 4.5%; Moisture not more than 12.0%, and Ash not more than 8.0%.

Ingredients: Dehulled Soybean Meal, Ground Corn, Wheat Middlings, Ground Wheat, Soybean Oil, Cane Molasses, Ground Oats, Calcium Carbonate, Dried Plain Beet Pulp, Dehydrated Alfalfa Meal, Wheat Germ, Brewers Dried Yeast, Salt, Calcium Propionate (a Preservative), Dicalcium Phosphate, Pyridoxine Hydrochloride, DL-Methionine, Choline Chloride, Dried Yucca Schidigera Extract, Menadione Sodium Bisulfite Complex (source of Vitamin K), Vitamin D3 Supplement, Vitamin A Acetate, Folic Acid, Thiamine Mononitrate, D-Alpha Tocopheryl Acetate, Vitamin B12 Supplement, Niacin Supplement, Calcium Pantothenate, Riboflavin, Manganous Oxide, Zinc Oxide, Ferrous Carbonate, Copper Sulfate, Zinc Sulfate, Calcium Iodate, Cobalt Carbonate.

Rodent High-fat Diet and Low-fat Diet Information: Certified ingredient composition can be found at Bio-Serv (S3282 and F4031).

High-fat Diet: Protein 20.5%; Fat 36%, and Carbonhydrate 35.7%.

Ingredients: Lard, Casein, Maltodextrin, Sucrose, Mineral Mix, Vitamin Mix, DL Methionine and Choline Chloride.

Low-fat Diet: Protein 20.5%; Fat 7.2%, and Carbonhydrate 61.6%.

Ingredients: Cornstarch, Sucrose, Casein, Maltodextrin, Lard, Mineral Mix, Vitamin Mix, DL Methionine, Choline Chloride.

**Surgery.** Roux-Y gastric bypass (RYGB) was performed as described in our previous reports [[1-3](#_ENREF_1)]. Surgical instrument was purchased from the Roboz, and sutures from the Ethicon (10-0 suture Cat# 2830G; 4-0 absorbable Cat# VR494, and 4-0 non-absorbable Cat# 1611G). All surgical preparations and procedures were approved by the University of Chicago Institutional Animal Care and Use Committee.

**References**

1. Yin DP, Boyd KL, Williams PE, Abumrad NN, Wasserman DH: **Mouse Models of Bariatric Surgery**. *Curr Protoc Mouse Biol* 2012, **2012**.

2. Yin DP, Gao Q, Ma LL, Yan W, Williams PE, McGuinness OP, Wasserman DH, Abumrad NN: **Assessment of different bariatric surgeries in the treatment of obesity and insulin resistance in mice**. *Annals of surgery* 2011, **254**(1):73-82.

3. Xu R, Zhu C, Pierre JF, Yin DP: **Gastric Bypass Improves Obesity and Glucose Tolerance Independent of Gastric Pouch Size**. *Obesity surgery* 2020, **30**(5):1635-1641.
