# Supplementary material for: Gastric bypass prevents diabetes in genetically modified mice and chemically induced diabetic mice
Source: PLoS One. 2021 Oct 21;16(10):e0258942. doi: 10.1371/journal.pone.0258942 (PMC8530305; doi:10.1371/journal.pone.0258942)
Supplement: Attachment — Submitted filename: Response to reviewers_updated.docx [file pone.0258942.s002.docx]

| **Reviewer’s specific comments** | **Response to reviewer’s comments** |
| --- | --- |
| **Reviewer #1** |  |
| While this has not been extensively studied for RYGB in mice, it seems that GLP-1 is not essential for VSG… Also, in Table 1 it says db/db mice (BKS and B6) were only operated on at 6 weeks of age. However in Figure 5 These mice had surgery at 12 weeks of age. Please clarify. | Thanks for the comments. We have also noticed that metabolic benefits induced by RYGB are associated with increased GLP-1 expression, in which the mechanisms are unclear. We have recently found that GLP-1 expression is related with a different distribution of bile acids, proximately and distally, and increased GLP-1 may be associated with improved insulin secretion, which is under investigation.  Gastric bypass was performed at 6 weeks of age in genetically modified mice (BKS), and at the time when blood glucose was > 300mg/dL for two continuous days after STZ injection (B6). All mice were monitored for 12 weeks after surgery. |

**Reviewer 2**

| About the introduction. The paper is original but very descriptive and there are many concerns about the conclusion. | First, we thank for the reviewer’s comments. This manuscript describes the importance of gastric bypass for the prevention of prediabetes progression in the mouse RYGB model. The hypothesis is intuitively clear, and the results are reliable. Based on the results that early intervention with RYGB improves hyperglycemia in genetically modified and chemically induced diabetic mice, we conclude that an early intervention by gastric bypass results in the better outcome in preventing overt diabetes. |
| --- | --- |
| Concerning mice rodent models:  -line 67: Indeed, the characteristic of gastric bypass surgery is to restore beta cell function quickly after the procedure and before any significant body weight loss. This has been established by Walter Pories in humans (1995)… How the authors may explain this difference of their procedure when compared to the literature? | As Dr. Pories states in their publication (1995), gastric bypass is now established as an effective and safe therapy for morbid obesity, which is associated with increased insulin secretion, our results have also shown improved metabolic phenotype and increased circulating insulin levels after RYGB, which is consistent with previous reports. Our recent studies suggest an important role of bile acids and their receptor, FXR, in reshaping the gut microbiota and regulating GLP-1; therefore, improving beta cell function and promoting insulin secretion independent of weight change (Obesity 2019;27:803-812, AJP-GLP 2016;311:G286). |
| -line 72: …… bariatric surgery has been performed by different teams in ob/ob mice and these papers showed that beta cell function recovery may be possible despite lack of effect of surgery on body weight. In addition, beta cells of ob/ob mice activated molecular pathways able to  reduce the deleterious effects of oxidative stress while db/db mice did not display such mechanisms. This explain why mean glucose levels are higher in db/db mice when compared to ob/ob mice. | We agree with reviewer’s comment that beta cells of ob/ob mice activated molecular pathways able to reduce the deleterious effects of oxidative stress, while db/db mice may not display such mechanisms. This explain why mean glucose levels are higher in db/db mice when compared to ob/ob mice. We have also noticed a publication that bariatric surgery regulates insulin secretion via novel miRNA-gene interactions in the pancreatic islet during the resolution of diabetes in ob/ob mice (EBioMedicine. 2020). In addition, in ob/ob mice, a nonsense mutation in codon 105 changes an Arg residue to a stop codon causing premature truncation, resulting complete lack functional leptin although there are high levels of leptin mRNA, whereas db/db mice reveal a single autosomal recessive mutation in the leptin receptor gene, resulting in abnormal mRNA splicing. The defective leptin receptor leads to the over-production of extracellular leptin, but lack of intracellular leptin action. Thus, different mouse models may respond differently to gastric bypass (Int J Obes. 2015;39:798), and RYGB results in a different effect in improving metabolism in the different mouse models through different mechanisms. BKS mice reveal early onset of hyperglycemia and hyperinsulinemia or normal insulinemia, and an uncontrolled hyperglycemia or overt diabetes develops due to severe depletion of pancreatic beta-cells with aging, which provides a clinically relevant mouse model to test if gastric bypass can prevent disease progression from prediabetes to overt diabetes. |
| -line 89-90: … Thus, STZ rodent model is a model of diabetes lacking insulin without auto-immunity. This is important if we consider the potential proliferation of new beta cells from ductal cells after STZ administration and bariatric surgery, mechanism not possible when autoimmunity is present. This may change insulin secretion capacity. | Thanks for the comment. Yes, single high dose of STZ (in our model) results in severe destruction of beta cells and develops diabetes, which is widely used as T1D model and may be revised by islet transplantation (our previous reports, AJT 2010;10:1588, Diabetes 2006;55:3256, Science 2006;311:1744…). While humoral response is revealed, STZ induces hyperglycemia in nude mice, indicating an T cell independent response. Previous reports and our study show that gastric bypass promotes secretion of insulin, and underlying mechanism may be related with increased expression of GLP-1. We hypothesize that gastric bypass mediated metabolic benefit may be associated increases of bile acids in the distal intestine and a lack of bile acids in the proximal intestine, which reshape composition of the gut microbiota and regulate metabolic pathways. |
| Results  -line 177: the figure 1B show that decrease of body weight is similar in RYGB and SHAM-PF db/db mice. I don’t understand line 179 the p< 0.05. | We state in line 177 that “The results in Fig 1b show that RYGB decreased body weight, and SHAM-PF experienced similar weight changes in BKS-db/db mice (BKS-db/db-SHAM-PF vs. BKS-db/db-RYGB, *p* > 0.05, Fig 1b)”. |
| -line 180 figure 1c: db/db mice have higher amount of fat mass than lean. there is no difference between fat mass in Sham-PF and RYBG groups. Is it correct ? | Yes, “there is no difference between fat mass in Sham-PF and RYBG groups.” |
| -line 186: glucose tolerance in RYGB is improved in comparison to control after the first two weeks of follow-up. The figure 2B showed a progressive increase of AUC glucose in RYGB mice suggesting a progressive deterioration of glucose homeostasis.. This has to be discussed. | Glucose tolerance is improved in RYGB compared to control mice, and this improvement is persistent to the end point of the experiment (12 weeks), which have been discussed in the Discussion. *: BKS-db/db-RYGB vs. BKS-db/db-Control and BKS-db/db-SHAM-PF, *p* < 0.001, through the 12^th^ weeks (Fig 2b). |
| -line 183: an absolute level of insulin without the glucose levels cannot be correctly analyzed. It is mandatory to plot glucose and insulin levels (or AUC glucose). There are no details about the metabolic status of mice (fasted or random fed). | We analyzed circulating insulin levels in untreated young, adult and RYGB treated BKS mice at the same time point, which is consistent with blood glucose levels described in Fig 2. All blood glucose tests and GTT were performed in mice with 4 hours fast, which have been described in the Methods. An additional explanation has been added (line 217). |
| -line 19: figure 3B : on this figure, the significant difference between young controls and adult controls has to be added. | Thanks for the comment, and the significant difference between young controls and adult controls has been added in the revised manuscript. |
| -line 205: figure 3c showed representative pancreata images. This is not sufficient and quantification of insulin + cells has to be showed and expressed by the weight of pancreas (because it cannot be excluded that surgery may modify the total weight of pancreas by changes in exocrine function). It is the same for figure 4c. | We realized that the accounting of insulin+ cells must be combined with pancreatic volume. Originally, we quantify beta cell area using IHC. Slides with 5 um-thick paraffin pancreas sections were deparaffinized and incubated with anti-Insulin antibody. The Bond Intense R detection system was used for visualization. Seven sections per pancreas were obtained at 250um intervals through the pancreas. These slides were scanned using the Aperio ScanScope CS bright-field slide scanner (Aperio Technologies, Inc., Vista, CA). The resulting images were used to quantify the percent of insulin-positive tissue area using the Genie function of Aperio’s Spectrum software. However, we realize that animal numbers (only 3 per group) are not sufficient to perform statistical analysis, and we did not use the data of cell counting and keep the imaging in the original submission. Thanks for the comment that imaging alone is not enough to show beta cell mass, we remove Fig 3c and Fig 4c in the revised manuscript. |
| -figure 4 and figure 5: all the figures with glucose levels during the follow-up or the IPGTT (figure 5b) needed the insulin secretion levels. It is difficult to conclude ... Extrapolation of in vivo insulin secretion from immunostaining is impossible and such extrapolation deserves the paper. | We concord with reviewer’s comment that the analysis of blood glucose and glucose tolerance should combine with insulin content. But practically, collection of blood samples for insulin analysis every time in performing blood glucose test is tough and difficult (at least once or twice a week). We also agree that “Extrapolation of in vivo insulin secretion from immunostaining is impossible…”, thus we removed Fig 3c and Fig 4c (with insulin+ cell imaging). |
| Thus, in vivo insulin secretion has to be studied and showed in this paper. In addition, the SOS study has showed in humans that gastric bypass may reduce incidence of T2D in non diabetic obese patients during a long-term follow-up suggesting that the prevention of diabetes is possible in humans. Thus, the present paper does not add novelty in this field. | While gastric bypass may reduce incidence of T2D in humans, we do not know if early surgery can result in a better outcome, and we do not know why gastric bypass can only effectively improve obesity and insulin resistance, complete resolution of diabetes occurs in 42.3 to 59.4% of patients. Likewise, less favorable outcomes may be associated with longer duration of diabetes, poor preoperative hyperglycemia control and reduced beta cell function. Our data suggest an early surgical intervention like gastric bypass in obese patients may be a viable strategy to prevent the progression of diabetes. The mechanistic study including the role of bile acids and the gut microbiota in the regulation of metabolism is under investigation. |

If you have any questions concerning this manuscript, please don't hesitate to contact me at the following addresses.

Thank you very much in advance for your consideration.

Dengping Yin, MD, PhD

Research Associate Professor

Director, Animal Microsurgery Center

Department of Surgery

The University of Chicago

Tel: 773-702-5520

email: dyin@surgery.bsd.uchicago.edu
